# 中国霍奇金淋巴瘤的诊断与治疗指南（2022年版）

**DOI:** 10.3760/cma.j.issn.0253-2727.2022.09.001

**Published:** 2022-09

**Authors:** 中国抗癌协会血液肿瘤专业委员会 中华医学会血液学分会 中国霍奇金淋巴瘤工作组

霍奇金淋巴瘤（Hodgkin lymphoma，HL）是一种少见的累及淋巴结及淋巴系统的恶性肿瘤。随着对疾病认识的加深及新药的临床应用，中国HL患者治疗选择增加，生存得到改善。为提高我国HL诊断、鉴别诊断及规范化治疗水平，中国抗癌协会血液肿瘤专业委员会、中华医学会血液学分会、中国霍奇金淋巴瘤工作组组织专家根据国际上相关指南及循证医学研究结果，结合目前我国淋巴瘤的诊治水平和现状制订了中国HL诊断与治疗指南（2022年版）。

一、定义

HL（旧称霍奇金病）是一种少见的累及淋巴结及淋巴系统的恶性肿瘤。HL分为结节性淋巴细胞为主型HL（nodular lymphocyte predominant Hodgkin lymphoma，NLPHL）和经典型HL（classic Hodgkin lymphoma，cHL）。cHL约占HL的90％，特征为肿瘤细胞-里德-斯特恩伯格（Hodgkin Reed-Sternberg，HRS）细胞与异质性非肿瘤炎性细胞混合存在，HRS细胞CD30高表达且下游NF-kappaB通路持续性激活，为青年人中最常见的恶性肿瘤之一[1]。cHL可分为4种组织学亚型，即结节硬化型、富于淋巴细胞型、混合细胞型和淋巴细胞消减型[2]。

全球数据（GLOBOCAN 2020）显示年全球新发HL共83 087例，其中男性48 981例，女性34 106例，死亡23 376例，其中男性14 288例，女性9 088例。而中国2020年新发HL也达6 829例，其中男性4 506例，女性2 323例，死亡2 807例，其中男性1 865例，女性942例[3]–[4]。在我国，HL占全部淋巴瘤的8.54％[5]，男性多于女性。我国HL发病年龄较小，中位发病年龄为30岁左右，90％的HL以淋巴结肿大为首发症状，以颈部淋巴结和锁骨上淋巴结常见，然后扩散至其他淋巴结，晚期可侵犯血管，累及脾、肝、骨髓和消化道等。

二、诊断、鉴别诊断、分期和预后

（一）病理诊断

1. 病理检查：病理检查是确诊及分型的金标准，推荐病变淋巴结或结外病灶切除活检，应选择增长迅速、饱满、质韧的肿大淋巴结，尽量完整切除；尽量选择受炎症干扰较小部位的淋巴结；术中应避免挤压组织，切取的组织应尽快切开固定。除切除活检外，不推荐细针穿刺细胞学检查，对于纵隔或深部淋巴结可以考虑行粗针多条组织穿刺活检以明确病理诊断。

2. 形态学：cHL有独特的病理特征，在炎症细胞和反应性细胞所构成的微环境中散在分布少量Reed-Sternberg（R-S）细胞及变异型R-S细胞。典型R-S细胞为体积大、胞质丰富，双核或多核巨细胞，核仁嗜酸性，大而明显；若细胞表现为对称的双核时则称为镜影细胞。NLPHL中典型R-S细胞少见，肿瘤细胞因细胞核大、折叠，似爆米花样，故又称为爆米花细胞或淋巴细胞性和（或）组织细胞性R-S细胞变型细胞。

3. 免疫组织化学评估：诊断HL应常规进行免疫组织化学评估，IHC标志物包括CD45（LCA）、CD20、CD15、CD30、PAX5、CD3、MUM1、Ki-67和EBV-EBER。cHL常表现为CD30（+）、CD15（+/−）、PAX5（弱+）、MUM1（+）、CD45（−）、CD20（−/弱+）、CD3（−）、BOB.1（−）、OCT2（−/+），部分患者EBV-EBER（+）。NLPHL常表现为CD20（+）、CD79α（+）、BCL6（+）、CD45（+）、CD3（−）、CD15（−）、CD30（−）、BOB1（+）、OCT2（+）、EBV-EBER（−）。在进行鉴别诊断时，需增加相应的标志物。

（二）鉴别诊断

很多情况都可引起淋巴结肿大，可能伴有发热、盗汗、体重减轻或其他表现。鉴别诊断包括感染性、自身免疫性和多种恶性疾病。

1. 反应性疾病：感染性、自身免疫性和其他炎性疾病均可引起淋巴结肿大、器官肿大、发热和其他难以与cHL区分的全身症状。反应性疾病可以出现类似cHL的多形性细胞浸润，但无诊断性HRS细胞，上述细胞可通过独特的形态和免疫表型确定。

2. EB病毒（EBV）阳性的皮肤黏膜溃疡：EBV阳性的皮肤黏膜溃疡是以孤立性局限性溃疡病变为特征的疾病，通常见于老年人，有时发生于免疫抑制者。病变最常见于口咽部，但也可发生于皮肤或胃肠道；表现为多形性炎性浸润背景中混有散在的EBV感染的B细胞，可能包括一些形态和免疫表型与HRS细胞类似的细胞。该病与cHL的鉴别要点是其结外表现、良性病程、经常自行缓解及保守治疗效果极好。

3. 间变性大细胞淋巴瘤（ALCL）：可能与淋巴细胞消减型cHL（LDCHL）的某些变异型难以区分，部分ALCL可产生炎性反应和组织纤维化，与宿主对HRS细胞的反应类似。然而，结合形态学和免疫表型特征一般均可区分开：

（1）cHL：CD15阳性、CD30阳性、PAX/BSAP阳性、T细胞抗原阴性，ALK阴性。

（2）ALCL：CD15阴性，CD30强阳性，PAX5/BSAP阴性，一种或多种T细胞抗原阳性，ALK阳性/阴性，细胞毒性标志物（穿孔素、颗粒酶B、TIA-1）阳性。

4. 其他B细胞淋巴瘤：

（1）原发性纵隔B细胞淋巴瘤（PMBL）：PMBL和结节硬化型cHL（NSCHL）有一些共同的临床特征，包括存在纵隔肿块及主要发生于年轻女性。PMBL的活检可能显示与cHL的HRS细胞类似的细胞，该病的基因表达模式与NSCHL相似。但PMBL的恶性细胞通常表达泛B细胞抗原，弱表达CD30，极少表达CD15。而cHL的HRS细胞通常表达CD15和CD30。HRS细胞表达的成束蛋白可帮助区分EBV阴性的cHL与PMBL。尽管如此，少数病例同时具有PMBL和HL的特征，属于灰区B细胞淋巴瘤，不能分类，其特征介于DLBCL和cHL之间。

（2）T细胞/组织细胞丰富型大B细胞淋巴瘤（THRLBCL）：THRLBCL也难以与cHL区分。THRLBCL最常见于中年男性，与cHL类似，肿瘤细胞可能仅占细胞总数的小部分（按照定义，肿瘤细胞比例小于10％）。然而，THRLBCL的恶性B细胞通常有类似于其他B细胞淋巴瘤的免疫表型，如泛B细胞标志物阳性，而CD15、CD30和EBV阴性。

（三）分期

淋巴瘤的临床分期依据疾病侵犯部位及有无B症状，目前采用的是2014版Lugano分期标准[6]（表1）。根据患者有无B症状［①不明原因发热>38 °C，连续3 d以上，排除感染；②夜间盗汗（可浸透衣物）；③体重于诊断前半年内下降>10％］分为A组（无B症状）和B组（有B症状）。

**表1:** 2014版淋巴瘤Lugano分期

分期	侵犯范围
局限期	
Ⅰ期	仅侵及单一淋巴结区域（Ⅰ期），或侵及单一结外器官不伴淋巴结受累（ⅠE期）
Ⅱ期	侵及横膈一侧≥2个淋巴结区域（Ⅱ期），可伴同侧淋巴结引流区域的局限性结外器官受累（ⅡE期）
Ⅱ期伴大包块	纵隔包块MMR>0.33，其他部位最大直径≥10 cm
进展期	
Ⅲ期	侵及横膈肌上下淋巴结区域，或横膈以上淋巴结区受侵伴脾脏受侵（ⅢS期）
Ⅳ期	侵及淋巴结引流区域外的结外器官

注：MMR：肿块最大径/胸腔最大径

（四）预后

预后评价主要分为局限期预后评分和进展期预后评分。Ⅰ～Ⅱ期cHL根据有无预后不良因素分为预后良好组及预后不良组（不良预后因素见表2），Ⅲ～Ⅳ期主要采用国际预后评分（IPS）（表3）。

**表2:** Ⅰ～Ⅱ期霍奇金淋巴瘤的不良预后因素

预后因素	EORTC	GHSG	NCCN
年龄	≥50岁		
ESR和B症状	>50 mm/1h且无B症状； >30 mm/1h且有B症状	>50 mm/1h且无B症状； >30 mm/1h且有B症状	≥50 mm/1h或有B症状
纵隔大肿块	MTR>0.35	MMR>0.33	MMR>0.33
受累淋巴结区	>3	>2	>3
结外病灶		有	
大肿块直径			>10 cm

注：EORTC：欧洲癌症研究与治疗组织；GHSG：德国霍奇金淋巴瘤研究组；NCCN：美国国立综合癌症网络；ESR：红细胞沉降率；MTR：肿块最大径/胸腔T5或T6水平横径；MMR：肿块最大径/胸腔最大径

**表3:** 晚期霍奇金淋巴瘤国际预后评分（IPS）

不良预后因素
白蛋白<40 g/L
血红蛋白<105 g/L
男性
年龄≥45岁
Ⅳ期病变
白细胞增多（WBC≥15×10^9^/L）
淋巴细胞减少［淋巴细胞计数小于白细胞总数的8％，和（或）淋巴细胞计数<0.6×10^9^/L］

注：表中每项因素计1分，积分0～3分为预后好，积分≥4分为预后差

三、治疗

HL患者疾病治愈的可能性很高，治疗的选择必须权衡取得高治愈率与尽量减少远期并发症。

（一）治疗前评估

治疗前（包括复发患者治疗前）应对患者进行全面评估，应至少包括：

1. 病史采集和体格检查：病史（包括有无B症状，淋巴结肿大的范围和持续时间，有无瘙痒、乏力、腹胀/腹痛及酒精不耐受）和体格检查（应评估肿大淋巴结的大小、数量和具体区域，有无肝肿大或脾肿大，心脏和呼吸系统状况及体能状态）。

2. 实验室检查：全血细胞计数、红细胞沉降率（ESR）、肝功能、肾功能、乳酸脱氢酶（LDH）、C反应蛋白（CRP）、碱性磷酸酶（ALP）、白蛋白；乙型肝炎病毒（HBV）表面抗原/抗体和核心抗体、HBV DNA及丙型肝炎病毒（HCV）、HIV；妊娠试验（针对育龄期女性）。

3. 心脏功能：通过超声心动图或放射性核素心室造影评估左室射血分数（LVEF）。若考虑使用以蒽环类药物为基础的化疗，则LVEF通常应≥50％。

4. 肺功能测定：若考虑使用含博来霉素的化疗方案（如ABVD方案或BEACOPP方案），有条件者可行肺功能测定（pulmonary function test，PFT），包括肺一氧化碳弥散量（diffusing capacity of the lungs for carbon monoxide，DLCO）。通常情况下，DLCO≥60％的患者可以使用博来霉素治疗。

5. 影像学检查：包括正电子发射计算机断层扫描（PET/CT）、全身增强CT、胸部X线。鼓励行胸部X线检查，尤其是在有较大纵隔肿物时。增强CT扫描范围为颈部/胸部/腹部/盆腔，至少应包括PET/CT检查显示异常的区域。PET/CT扫描前患者禁食6～8 h以上，测患者血糖（≤11.1 mmol/L）。静息坐卧15 min后注射^18^F-FDG（3.7～7.4 mBq/kg），封闭视听神经静卧（60±5）min，排空膀胱并饮水后，行常规PET/CT扫描。扫描范围为颅顶至中部大腿（必要时加做四肢扫描）。应用CT数据进行衰减校正，获得全身PET图像、CT图像及PET/CT融合图像，所有图像通过工作站显示。在特定病例中需要加做增强MRI或PET/MRI。

6. 骨髓检查：待诊断患者可行骨髓穿刺和活检，若已行PET/CT检查，则可不选择骨髓检查。如果存在贫血以外无法解释的血细胞减少（如血小板减少或中性粒细胞减少）和PET/CT阴性，则进行充分的骨髓活检。

（二）治疗方案

1. 初治cHL的一线治疗：HL的治疗目标为治愈，同时在不影响疗效的情况下尽可能减轻治疗相关毒性反应，降低早期及晚期并发症发生风险[7]。HL应采用综合治疗及个体化治疗的原则，依据分期及有无预后不良因素进行分层治疗，Ⅰ～Ⅱ期HL采用以化疗联合放疗为主的综合治疗，单纯化疗适用于部分放疗长期毒性风险超过疾病短期控制获益的患者。Ⅲ～Ⅳ期cHL的治疗原则通常为化疗，局部放疗仅限于化疗后残存病灶超过2.5 cm者。对于早期患者应追求更低的毒性，减少合并症，降低继发性肿瘤风险，降低心脏及肺脏毒性，而晚期患者应设法提高治愈率。

Ⅰ～ⅡA期预后良好患者的治疗：标准治疗为2～4个周期ABVD方案（阿霉素＋博来霉素＋长春花碱＋达卡巴嗪）化疗联合放疗。对于一部分不伴危险因素、预后良好的患者可行ABVD×2个周期+放射治疗（RT）（20 Gy）[8]；也可以根据ABVD×2个周期后PET/CT评估结果调整用药方案（图1）[9]，但即使早期PET/CT阴性，综合治疗的无进展生存（PFS）率也较单纯化疗更高。

**图1:**
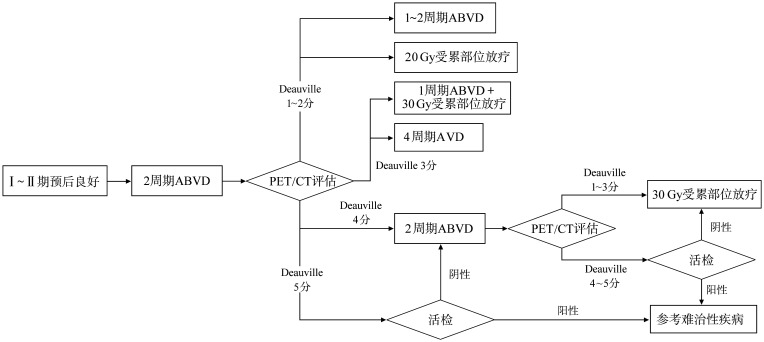
Ⅰ～ⅡA期预后良好霍奇金淋巴瘤患者的治疗 ABVD：阿霉素＋博来霉素＋长春花碱＋达卡巴嗪；AVD：阿霉素＋长春花碱＋达卡巴嗪

Ⅰ～ⅡB期预后不良患者的治疗：标准治疗为2个周期ABVD方案后行PET/CT评估，评分1～3分患者考虑2个周期ABVD方案化疗联合30 Gy放疗或4个周期AVD方案；评分4～5分的患者推荐2个周期增强剂量BEACOPP方案后再行PET/CT评估[10]，根据PET/CT评估结果调整用药方案[9]（图2）。

**图2:**
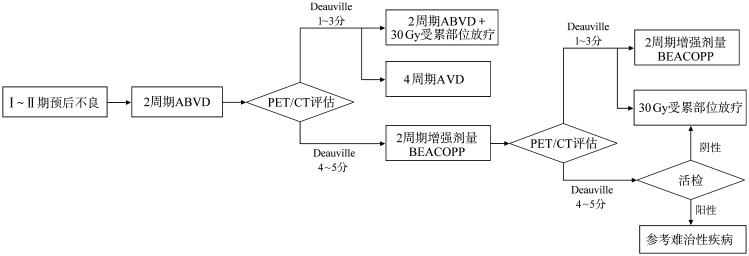
Ⅰ～ⅡB期预后不良霍奇金淋巴瘤患者的治疗 ABVD：阿霉素＋博来霉素＋长春花碱＋达卡巴嗪；AVD：阿霉素＋长春花碱＋达卡巴嗪；BEACOPP：博来霉素+依托泊苷+阿霉素+环磷酰胺+长春新碱+丙卡巴嗪+泼尼松

Ⅲ～Ⅳ期患者的治疗：标准治疗为ABVD方案×6个周期±RT[11]，局部放疗仅限于化疗后残存病灶2.5 cm以上者，期间行PET/CT评估，评分4～5分患者更换高强度方案化疗；对于≤60岁患者，增强剂量BEACOPP方案×4～6个周期可提高PFS率，但骨髓抑制、生殖系统不良反应和第二原发肿瘤累积发生率增加[12]–[13]；根据ABVD方案/增强剂量BEACOPP方案×2个周期后PET/CT评估结果调整用药方案（图3）。另外，基于2021年公布的ECHELON-1研究结果，BV联合AVD组和ABVD组5年改良PFS率分别为82.2％和75.3％（*P*＝0.0017），达到研究终点，同时改善了肺毒性，推荐BV+AVD方案×6个周期[14]，用于初始Ⅲ～Ⅳ期cHL成年患者（图3）。

**图3:**
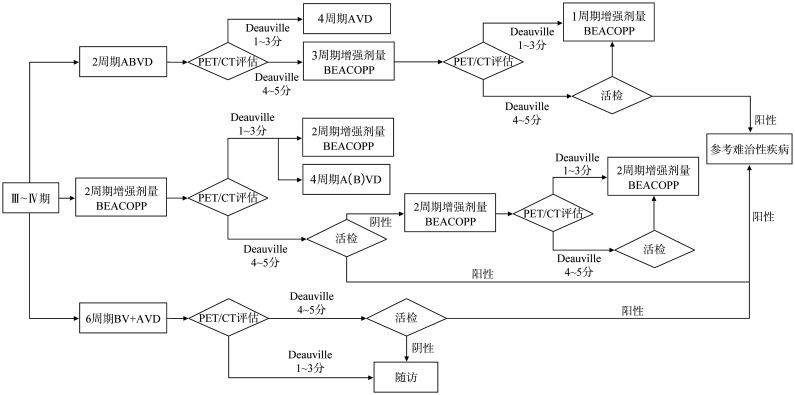
Ⅲ～Ⅳ期霍奇金淋巴瘤患者的治疗 ABVD：阿霉素＋博来霉素＋长春花碱＋达卡巴嗪；AVD：阿霉素＋长春花碱＋达卡巴嗪；BEACOPP：博来霉素+依托泊苷+阿霉素+环磷酰胺+长春新碱+丙卡巴嗪+泼尼松；BV：维布妥昔单抗

值得注意的是，ABVD方案中的长春花碱目前国内不可及，可用其他长春碱类药物替代，如长春地辛（3 mg/m^2^）。鉴于eBEACOPP方案中药物的可及性及相对较大的近期和远期不良反应[15]，各中心可根据自己的情况选择合适的方案。

2. 复发或难治性cHL的治疗方案：复发或难治性cHL的治疗目标主要有两个，一是采用优化的风险适应性治疗方案实现长期疾病控制（即治愈），二是根据复发低风险患者的大剂量化疗（HDT）/自体造血干细胞移植（ASCT）需求评估选择治疗方案，降低治疗相关毒性和并发症[16]。

复发或难治性cHL的治疗原则首选二线挽救方案化疗后进行大剂量化疗联合ASCT，二线化疗尽可能达到完全缓解（CR）。维布妥昔单抗联合化疗方案如ICE方案（CR率88％）[17]、ESHAP方案（CR率70％）[18]、DHAP方案（CR率81％）[19]使更高比例患者获得了CR，增加了ASCT的成功率。维布妥昔单抗联合苯达莫司汀（CR率73.6％）[20]及维布妥昔单抗联合PD-1单抗（CR率61％）[21]是NCCN（2022年版）指南的推荐联合用药方案[22]–[23]。PD-1单抗联合GVD方案（CR率85.2％）[24]，PD-1单抗联合ICE方案（CR率86.5％）[24]–[25]，PD-1单抗联合Gemox方案（CR率90％）[26]，在复发/难治性（r/r）HL中获得较好疗效。对于不符合ASCT条件的患者，可选择化疗、维布妥昔单抗±化疗、PD-1单抗±化疗和（或）放疗。挽救治疗方案见图4及表4。

**图4:**
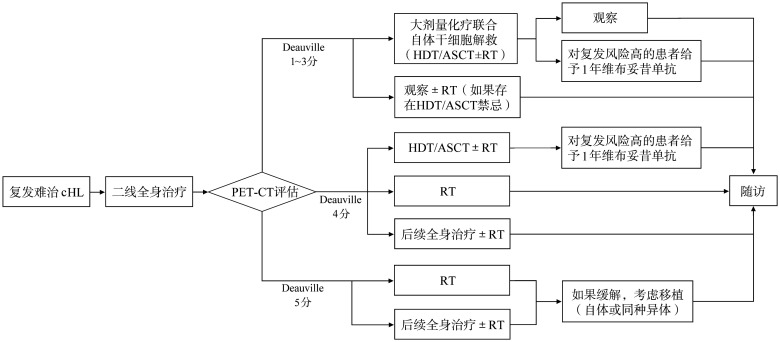
复发难治经典型霍奇金淋巴瘤（cHL）的治疗 HDT：大剂量化疗；ASCT：自体造血干细胞移植；RT：放射治疗

**表4:** 复发/难治性经典型霍奇金淋巴瘤的治疗方案

治疗方案	治疗方案
维布妥昔单抗	苯达莫司汀
维布妥昔单抗+苯达莫司汀	苯达莫司汀+卡铂+依托泊苷
维布妥昔单抗+PD-1单抗	C-MOPP（环磷酰胺+长春新碱+甲基苄肼+泼尼松）
维布妥昔单抗+ICE或ESHAP或DHAP方案	依维莫司
DHAP方案（地塞米松+顺铂+大剂量阿糖胞苷）	GCD（吉西他滨+卡铂+地塞米松）
ESHAP方案（依托泊苷+甲泼尼龙+顺铂+大剂量阿糖胞苷）	GEMOX（吉西他滨+奥沙利铂）
GDP方案（吉西他滨+顺铂+地塞米松）	来那度胺
GVD方案（吉西他滨+长春瑞滨+脂质体阿霉素）	MINE（依托泊苷+异环磷酰胺+美司钠+米托蒽醌）
ICE方案（异环磷酰胺+卡铂+依托泊苷）	Mini-BEAM（卡莫司汀+阿糖胞苷+依托泊苷+美法仑）
IGEV方案（异环磷酰胺+吉西他滨+长春瑞滨）	PD-1单抗
卡瑞利珠单抗+地西他滨	PD-1单抗+GVD或ICE或苯达莫司汀或GEMOX

移植后巩固：对于接受ASCT且移植后复发风险较高的患者，维布妥昔单抗维持治疗可以延长患者PFS时间，我们建议ASCT后30～45 d开始维布妥昔单抗治疗，1.8 mg/kg，每3周1次，最长持续16个周期。AETHERA是唯一针对移植后巩固治疗的研究，维布妥昔单抗治疗组的PFS期较安慰剂组显著延长（*HR*＝0.57，95％*CI* 0.40～0.81，*P*＝0.001），长达42.9个月[27]–[30]。PD-1单抗维持治疗可能对接受ASCT后的r/r cHL患者有临床效果，一项多中心研究在患者ASCT出院后的21 d内开始静脉予帕博利珠单抗治疗，20 mg/次，每3周1次，最长持续8个周期[31]。28例可评估患者的18个月PFS率为82％，总生存（OS）率为100％，毒性可控。维布妥昔单抗联合PD-1单抗也为可选方案[32]。

移植后或后续复发：维布妥昔单抗、PD-1单抗如信迪利单抗、替雷利珠单抗、卡瑞利珠单抗、帕博利珠单抗和赛帕利珠单抗可用于大剂量化疗联合ASCT后复发的患者[33]–[37]。抗体药物偶联物关键Ⅱ期研究显示，维布妥昔单抗单药治疗r/r HL的总体有效率为75％，CR率为34％，中位治疗周期数为9（1～16）个。PD-1单抗在r/r HL患者中的单药有效率为69％～90.6％，CR率为16％～62.9％[38]。PD-1单抗联合苯达莫司汀（CR率57％）[33],[39]–[40]、卡瑞利珠单抗联合地西他滨（CR率71％）也是复发或难治性cHL患者的可选方案[33],[39]。ASCT后复发且仍对化疗敏感的年轻患者可考虑行异基因造血干细胞移植（allo-HSCT）治疗。使用维布妥昔单抗及免疫检查点抑制剂后复发或难治性患者首选进入临床试验，此外，常规联合化疗桥接allo-HSCT可选。不符合allo-HSCT条件患者可考虑采用单药姑息化疗方案，挽救放疗在其他治疗方案受限的情况下可考虑采用[41]。

3. NLPHL的治疗：无临床不良预后因素的Ⅰ～ⅡA期患者首选单纯放疗（30 Gy）[42]。ⅠB～ⅡB期或有临床不良预后因素的Ⅰ～ⅡA期患者可采用化疗±利妥昔单抗±放疗。Ⅲ～Ⅳ期根据临床判断采用化疗±利妥昔单抗±放疗或利妥昔单抗，化疗方案可选择ABVD、CHOP、CVP方案。治疗采用PET/CT评估，如缓解且无症状则观察，以前未行RT可考虑RT，如疾病稳定或进展则需进行活检，活检结果阴性且无症状可继续观察，活检结果阳性则参考以下复发难治患者治疗方案。

NLPHL复发难治性患者：对疑似复发者推荐重复PET/CT或诊断性CT评估，再重新进行活检以排除转化为侵袭性B细胞淋巴瘤的可能。复发时病变局限者可应用利妥昔单抗单药治疗，病灶广泛者可选择利妥昔单抗联合二线挽救方案治疗。

4. 老年HL患者：老年cHL患者常伴有不良结局，其中有B症状、体能状态差、混合细胞型、淋巴细胞耗竭型、EBV阳性和有合并症患者更为常见。老年患者相关研究数据较少，因此在标准和替代一线治疗之间的选择应基于临床判断，以取得最高疗效且尽量降低毒性为目标。

Ⅰ～Ⅱ期预后良好型：首选A（B）VD方案×2个周期±AVD方案×2个周期+受累部位放疗（ISRT）（20～30 Gy）。

Ⅰ～Ⅱ期不良病变或Ⅲ～Ⅳ期病变：A（B）VD方案×2个周期+AVD方案×4个周期，ABVD方案2个周期治疗后PET/CT阳性患者需要个体化治疗。对于治疗有效的患者（CR或部分缓解），维布妥昔单抗继以AVD方案，有条件应予维布妥昔单抗治疗增加患者获益。也可考虑维布妥昔单抗+DTIC（达卡巴嗪）方案[43]–[44]。

复发或难治性老年患者的结局普遍较差，无法作出统一建议，但可推荐临床试验或可能的单药治疗联合姑息疗法，选择包括：苯达莫司汀、维布妥昔单抗、RT、PD-1单抗。

5. 放疗原则以及剂量推荐[45]：根据临床状况，可采用光子或质子治疗，化疗后采用受累部位或受累淋巴结照射，不做扩大野或大面积照射。建议使用调强放疗（IMRT）技术，为了减少心脏照射，可采用呼吸门控技术（DIBH）等放疗新技术。

ISRT或受累淋巴结放疗（INRT）剂量如下：

（1）联合治疗：

①非肿块型病变（Ⅰ～Ⅱ期）：20～30 Gy（若采用ABVD方案），每次1.8～2.0 Gy；

②非肿块型病变（ⅠB～ⅡB期）：30 Gy，每次1.8～2.0 Gy；

③肿块型病变部位（所有分期）：30～36 Gy，每次1.8～2.0 Gy；

④对化疗部分缓解的部位（Ⅰ～Ⅱ期）：30～36 Gy，每次1.8～2.0 Gy。

（2）单纯ISRT（不常用，可用于早期NLPHL）：

受累区：30～36 Gy（NLPHL患者主要采用30 Gy），每次1.8～2.0 Gy；

非受累区：25～30 Gy，每次1.8～2.0 Gy；

ISRT用于NLPHL时，临床靶区（CTV）比化疗后大，建议包括整个受累的淋巴结区域。

（3）姑息放疗：4～30 Gy。

6. HL治疗相关并发症的治疗：HL化疗应关注剂量相关不良反应，针对不良反应给予支持治疗。

在应用维布妥昔单抗时，应重点监测周围神经病变（PN）和中性粒细胞减少症，若PN≥2级需暂停给药，直至毒性恢复到≤1级或基线水平，重新开始治疗需考虑将剂量降至1.2 mg/kg；若出现4级，则终止治疗。对于出现3级或4级中性粒细胞减少症的患者，需暂停给药，直至毒性恢复至≤2级或基线水平，然后采用相同剂量的给药方案重新开始治疗。在后续周期中考虑使用粒细胞集落刺激因子（G-CSF）或粒细胞-巨噬细胞集落刺激因子（GM-CSF）支持治疗。维布妥昔单抗联合AVD方案初始治疗cHL时，建议预防性使用G-CSF。

标准治疗方案中的博来霉素相关肺毒性会影响HL患者的OS，其中接受ABVD方案治疗的患者中有25％的患者会出现博来霉素相关肺毒性，而发生博来霉素肺毒性患者的5年OS率较不发生博来霉素肺毒性患者降低27％，肺毒性反应表现为呼吸困难、咳嗽、胸痛、肺部啰音等，导致非特异性肺炎和肺纤维化，患者甚至快速死于肺纤维化。HL患者的基线检查应包括肺功能检查，其中必须含DLCO指标，并建议所有患者戒烟。有研究显示，通常基线DLCO≥60％的HL患者可以使用含博来霉素的化疗方案进行治疗[46]。另外，在使用含博来霉素的化疗方案治疗期间，建议至少每4个周期评估1次肺功能，并根据结果决定患者是否能继续应用含博来霉素的化疗方案治疗。

第二肿瘤的发生：HL生存者的第二恶性肿瘤大多为实体瘤，其中乳腺癌、肺癌和胃肠道癌最常见。尽管总体上血液系统恶性肿瘤较少见，但相对危险度高于一般人群，相对风险：白血病：10～80倍；非霍奇金淋巴瘤（NHL）：3～35倍；实体瘤（肺癌、乳腺癌、骨癌、胃癌、结肠癌、甲状腺癌及黑色素瘤）：大于2倍。

心血管疾病：心血管疾病是HL长期幸存者中最常见的非恶性肿瘤致死原因。HL治疗后可能出现冠状动脉疾病、瓣膜病变、心包疾病、心律失常、心肌病和外周动脉疾病。

生育力的影响：几乎所有男性患者在接受含烷化剂化疗方案治疗后都会发生无精子症，后期精子活力恢复的比例极低，而ABVD方案也可致男性患者短期无精子症发生[47]。女性HL患者在接受烷化剂化疗后，可能出现卵巢功能减退，提早绝经及发生闭经的比例都显著升高[48]。建议在拟行治疗前，进行生育咨询，行保留生育功能治疗方案，包括低温保存精液、体外受精（IVF）或低温保存卵巢组织或卵母细胞。

四、疗效评价

HL的疗效评价主要参考2014年Lugano疗效评价标准[6]，推荐应用PET/CT或全身增强CT扫描检查评估（表5）。PET/CT采用Deauville评分系统（表6）进行评估[49]。肿瘤免疫治疗（尤其是免疫检查点抑制剂治疗）疗效评估标准推荐使用LYRIC（lymphoma response to immunomodulatory therapy criteria）标准（表7）[50]。

**表5:** 2014 Lugano疗效评价标准

疗效	部位	PET/CT（代谢缓解）	CT（影像学缓解）
完全缓解	淋巴结和结外病灶	Deauville评分法“1～3分”，伴或不伴残余肿块	靶病灶（淋巴结）LDi≤1.5 cm且无淋巴结外病灶
	不可测病灶	不适用	消失
	器官肿大	不适用	恢复正常
	新发病灶	无	无
	骨髓	骨髓无^18^F-FDG亲和性病灶证据	形态正常，如不能明确需流式细胞术检查阴性
部分缓解	淋巴结和结外病灶	Deauville评分法“4或5分”，^18^F-FDG摄取较基线降低，残余病灶可为任意大小	最多6个靶病灶SPD降低≥50%，如病灶过小，CT无法测量，5 mm×5 mm为默认值，不可见病灶为0 mm×0 mm
	不可测病灶	不适用	消失/正常，残余病灶未增大
	器官肿大	不适用	脾脏垂直径缩小值>原垂直径增大值的50%
	新发病灶	无	无
	骨髓	残留摄取高于正常骨髓组织但较基线降低。如淋巴结缓解情况下骨髓持续存在结节性异常改变，需MRI或活检进一步诊断	不适用
无缓解或疾病稳定	淋巴结和结外病灶	Deauville评分法“4或5分”，在中期或治疗结束时FDG摄取与基线比较无显著变化	最多6个靶淋巴结SPD降低<50%，且不符合疾病进展所有条件
	不可测病灶	不适用	没有与进展相符的增加
	器官肿大	不适用	没有与进展相符的增加
	新发病灶	无	无
	骨髓	与基线相比无变化	不适应
进展性疾病	单独的靶病灶（淋巴结/结节性肿块、结外病灶）	Deauville评分法“4或5分”，^18^F-FDG摄取较基线增加，和（或）与中期或治疗结束评估相一致的新发高FDG摄取病灶	至少一个靶病灶进展即可诊断，淋巴结/结外病灶需同时符合下述要求：①LDi>1.5 cm；②PPD最低点相比增加≥50%（较最小状态）；③LDi或SDi较最小状态增加0.5 cm（≤2 cm病灶）或1 cm（>2 cm病灶）
	器官肿大	不适用	脾大情况下，脾脏垂直径增大>原垂直径增大值的50%；若基线无脾大，垂直径需在基线基础上至少增加2 cm；新发或复发脾脏肿大
	不可测病灶	无	新发或不可测量病灶明确进展
	新发病灶	新发^18^F-FDG亲和性病灶与淋巴瘤一致，病因不明确时应行活检	新发淋巴结任意径线>1.5 cm；新发结外病灶任意径线>1 cm，如<1 cm，必须能够证实与淋巴瘤相关；明确与淋巴瘤相关的任意大小的病灶
	骨髓	新发或复发^18^F-FDG亲和性病灶	新病灶或复发病灶

注：LDi：病灶最长径；SDi：垂直于LDi的病灶最短径；PPD：单个病灶LDi与SDi的乘积；SPD：多个病灶的PPD之和

**表6:** PET/CT 5分评分（Deauville标准）

评分		PET/CT检查结果
阴性	1	病灶^18^F-FDG摄取不超过背景放射性分布
	2	病灶的^18^F-FDG摄取≤纵隔血池
	3	纵隔血池<病灶的^18^F-FDG摄取≤肝血池
阳性	4	任何部位病灶的^18^F-FDG摄取相对于肝血池有轻度或中度增高
	5	任何部位病灶的^18^F-FDG摄取相对于肝血池有显著增高（SUVmax>2倍肝血池）或出现新发病灶
	X	新发病灶有^18^F-FDG摄取，但与淋巴瘤无关

**表7:** 免疫治疗疗效评估LYRIC标准

疗效	LYRIC标准
完全缓解	同Lugano标准
部分缓解	同Lugano标准
疾病复发或进展	同Lugano标准，但需除外以下不确定的缓解（indeterminate response，IR）情况： IR（1）：在12周内病灶SPD增加≥50%（基于6个可测量病灶的 SPD），临床无恶化； IR（2）：在治疗后任何时间点SPD增加<50%，但出现新病灶；治疗中一个或多个病灶PPD≥50%（基于6个可测量病灶的SPD），病灶数量未增多； IR（3）：病灶^18^F-FDG摄取增高，病灶大小未见变化

注：PPD：单个病灶最长径与最短径的乘积；SPD：多个病灶的PPD之和

五、随访监测

本指南所含的建议基于霍奇金淋巴瘤工作组的成员机构临床实践，尚无高水平的循证医学证据支持，考虑到治疗结束后随访及迟发反应监测对于评估患者疾病状态及发展情况十分重要，故给出以下推荐。

1. 应记录CR，包括治疗结束后3个月内PET/CT转阴。

2. 建议在患者治疗结束时向其提供治疗总结，包括RT、危及器官（OAR）和蒽环类药物累积剂量的详细信息。

3. 建议在治疗后的前5年内积极随访，以便发现复发及评估是否有迟发性反应，查体和血液学检查的频率推荐：第1～2年每3～6个月1次；第3～5年每6～12个月1次；之后每年1次。颈、胸、腹、骨盆CT频率：治疗结束后前2年不超过半年，之后根据情况。如果最近一次PET/CT提示评分为4～5分，则需进行PET/CT检查，以确认CR。监测性PET/CT检查可增加假阳性风险，因此不常规使用。

4. 5年后随访和监测主要关注远期不良反应，特别关注第二肿瘤（肺癌、乳腺癌、急性髓系白血病、NHL）的发生及脏器损伤，包括肺、心脏、甲状腺及生育能力。第二肿瘤多发于治疗结束10年后，因此建议定期进行乳腺检查。颈部放疗者定期检查甲状腺功能和颈动脉超声。

六、结语与展望

HL的总体疗效及预后良好，但其诊疗也存在一些不足之处。中国幅员广阔，各地区发展不均衡，导致HL诊疗水平参差不齐。近年来，HL规范化诊疗在我国专业领域学者的努力下已有长足进步，如更加广泛地应用PET/CT作为评估分期的标准，另外，一些新药如CD30单抗、免疫检查点抑制剂的应用也取得了相应成果。本文从HL诊断治疗的角度，对不同分期分型的HL提供治疗方案共识性推荐，在新药应用方面力求为临床医师提供相应参考，进一步推动HL规范化诊疗发展，推动“健康中国2030规划”落地，提高患者的生存及生活质量。
